# Correction: Variability in the discharge of the Mississippi River and tributaries from 1817 to 2020

**DOI:** 10.1371/journal.pone.0316144

**Published:** 2024-12-17

**Authors:** The PLOS ONE Staff

## Notice of Republication

This article was republished on April 19, 2024, to correct mislocated text incorrectly placed in the conclusion during the typesetting process. The publisher apologizes for the errors. Please download this article again to view the correct version. The originally published, uncorrected article and the republished, corrected articles are provided here for reference.

## Supporting information

S1 File Originally published, uncorrected article. (PDF)

S2 File Republished, corrected article. (PDF)

## References

[1] Turner RE (2022) Variability in the discharge of the Mississippi River and tributaries from 1817 to 2020. PLoS ONE 17(12): e0276513. doi: 10.1371/journal.pone.0276513 36480558 PMC9731447

