# Supplementary figures and images for: Variability in the discharge of the Mississippi River and tributaries from 1817 to 2020
Source: PLoS One. 2022 Dec 8;17(12):e0276513. doi: 10.1371/journal.pone.0276513 (PMC9731447; doi:10.1371/journal.pone.0276513)

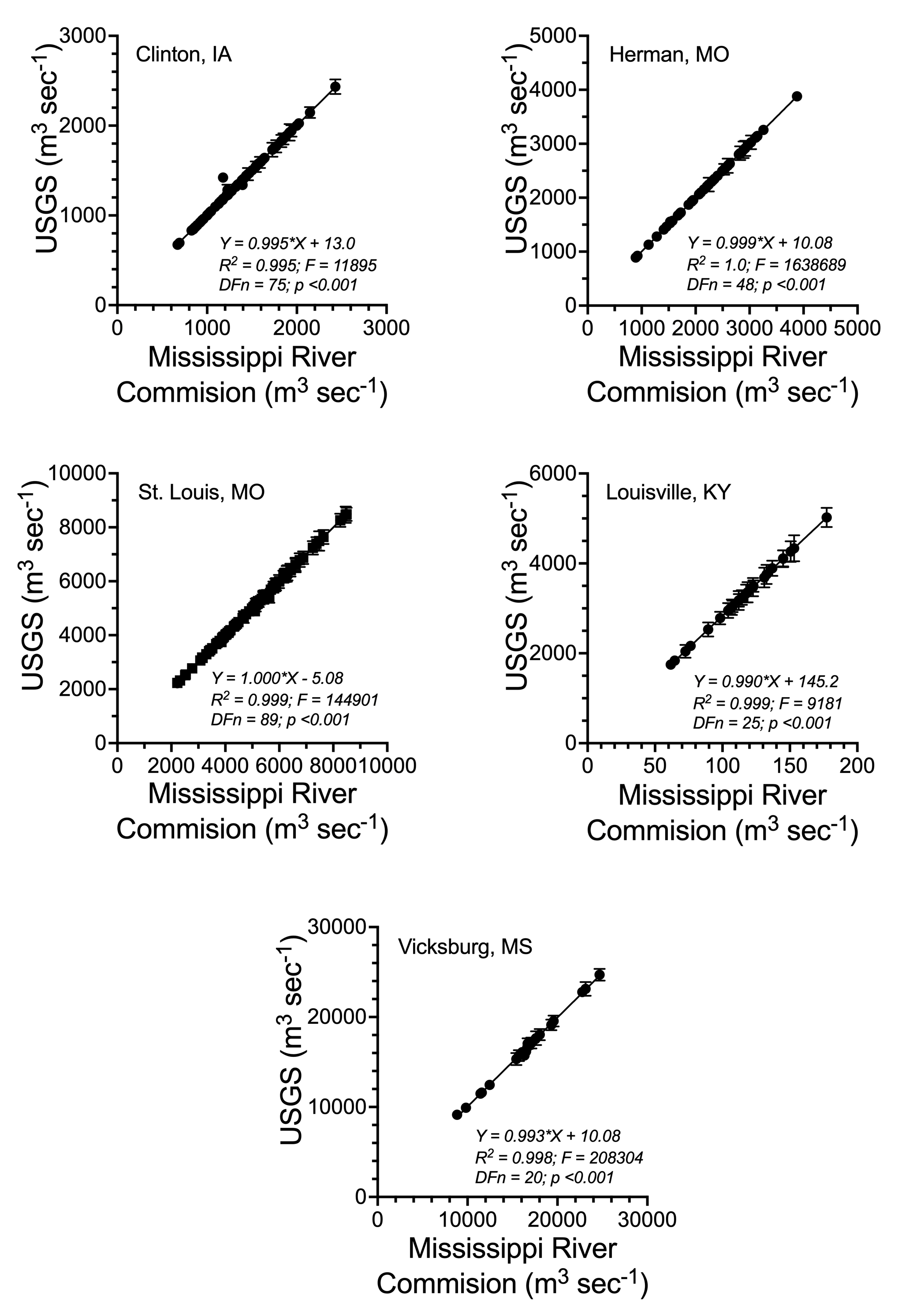

Supplement: S1 Fig — The number of overlapping years ranges from 20 to 90 years and the Coefficient of Determination (R2) = 0.99 and have a slope of 0.99 in all data sets. (TIFF) [file pone.0276513.s001.tiff]

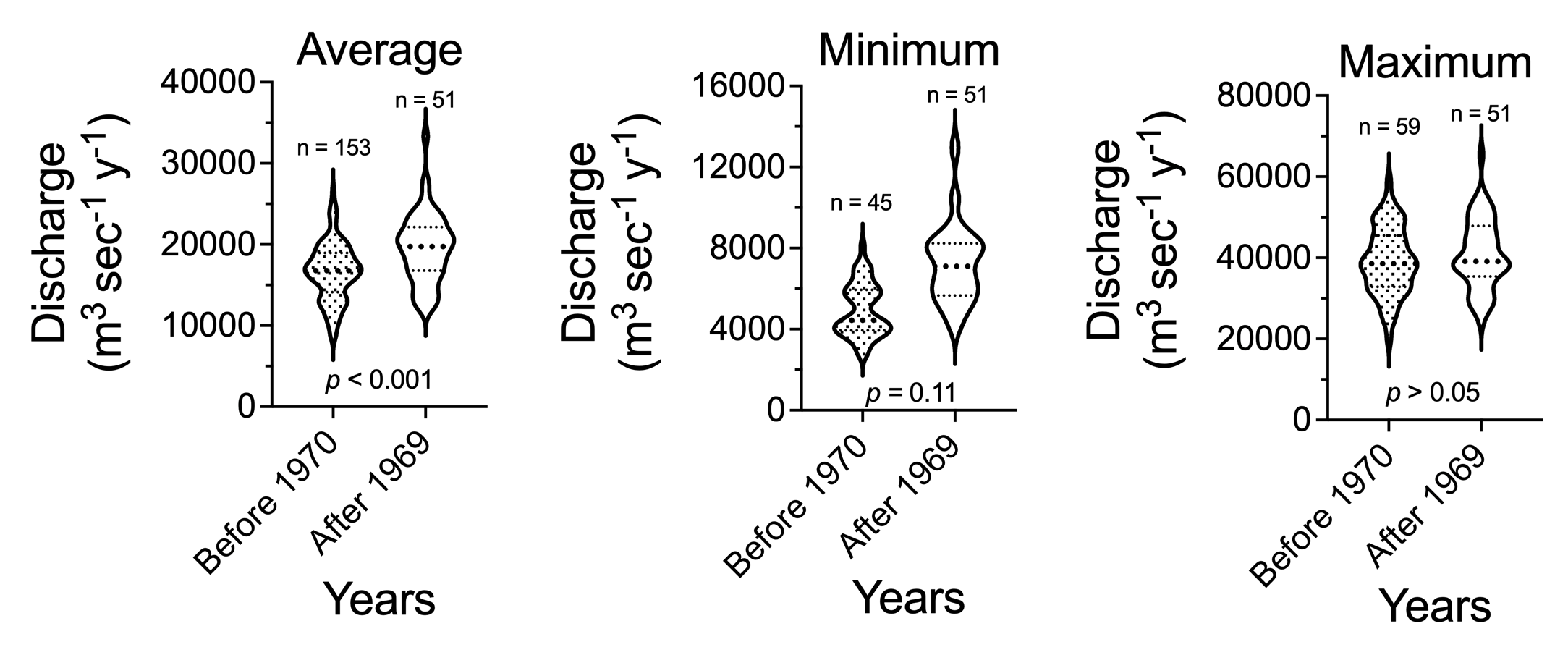

Supplement: S2 Fig — The mean and upper and lower quartiles are shown for each pair of sets. The probability of differences is shown for each of the three pairs. The t-test was for an unpaired parametric test with Welch’s correction: Average: F = 4.165, Dfd = 152; Minimum: F = 2.175, Dfd = 44; Maximum: F = 1.065, Dfd = 58. (TIFF) [file pone.0276513.s002.tiff]

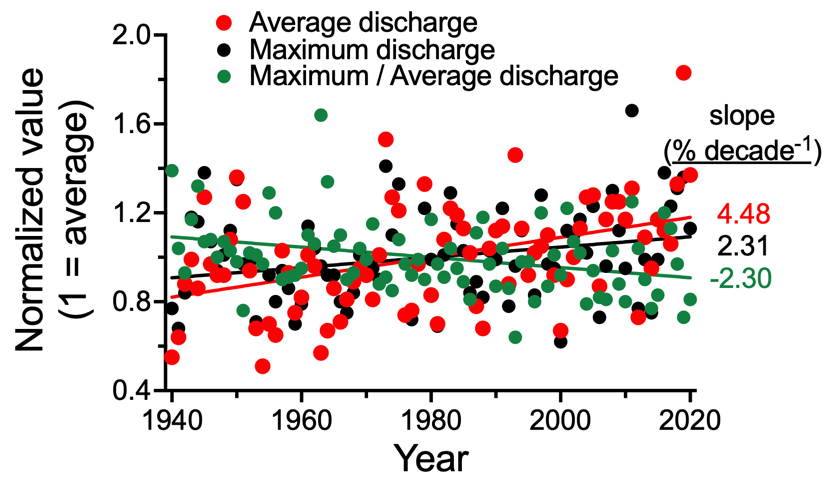

Supplement: S3 Fig — The equations are: 1) Average discharge: Y = 0.004483*X—7.876; F = 18.2, R2 = 0.19, p < 0.001; 2) Maximum discharge: Y = 0.002309*X—3.572; F = 5.47, R2 = 0.06, p = 0.02; 3) Maximum:Average: Y = -0.002297*X + 5.548; F = 9.72, R2 = 0.11, p < 0.01. The slopes are different from each other. The average discharge is rising at 4.48% decade-1, but the maximum discharge is rising 2.31% decade-1. The difference can be attributed to the lower amount of the average discharge that becomes a maximum discharge, probably because dams and reservoirs reduce the height of peak discharges. (TIFF) [file pone.0276513.s003.tiff]
