# Supplementary material for: Correction: Variability in the discharge of the Mississippi River and tributaries from 1817 to 2020
Source: PLoS One. 2024 Dec 17;19(12):e0316144. doi: 10.1371/journal.pone.0316144 (PMC11651566; doi:10.1371/journal.pone.0316144)
Supplement: S2 File — (PDF) [file pone.0316144.s002.pdf]

RESEARCH ARTICLE

Variability in the discharge of the Mississippi River and tributaries from 1817 to 2020

R. Eugene Turner *

Department of Oceanography and Coastal Sciences, Louisiana State University, Baton Rouge, Louisiana, United States of America

* euturne@lsu.edu

Check for updates

OPEN ACCESS

Citation: Turner RE (2022) Variability in the discharge of the Mississippi River and tributaries from 1817 to 2020. PLoS ONE 17(12): e0276513. https://doi.org/10.1371/journal.pone.0276513

Editor: Yougui Song, Institute of Earth and Environment, Chinese Academy of Sciences, CHINA

Received: March 7, 2022

Accepted: October 7, 2022

Published: December 8, 2022

Copyright: © 2022 R. Eugene Turner. This is an open access article distributed under the terms of the Creative Commons Attribution License, which permits unrestricted use, distribution, and reproduction in any medium, provided the original author and source are credited.

Data Availability Statement: The river discharge data assembled are available at the Dryad repository at https://doi.org/10.5061/dryad.1jwstqjzb.

Funding: RET received the NOAA National Centers for Coastal Ocean Science (https://coastalscience.noaa.gov/) MULtiSTRESS Award No. NA16OP2670 and NSF Award Number (FAIN) 2103843: Large-scale CoPe Hub: Rising Voices, Changing Coasts: The National Indigenous and Earth Sciences Convergence Hub. The funders had no role in study

Abstract

There are conflicting predictions of climate change effects and landuse on the discharge of the Mississippi River–the largest river in North America. Are discharges becoming higher or lower, and if they did change, then when? To address these uncertainties I compiled a two-hundred-year long dataset of the annual average, minimum, and maximum discharges at five stations draining the Mississippi River watershed: at Clinton, IA, Herman, MO, St. Louis, MO, Louisville, KY, and Vicksburg, MS. A spline/Lowess analysis tested for trends and inflection points. All three discharge metrics increased, and the minimum annual discharge increased faster than either the annual maximum discharge or annual average discharge. A regression analysis of variations in average discharges from 1950 to 2020 at these five locations demonstrates correlations to the air pressure differentials represented in the North Atlantic Oscillation (NAO) Index for January, February and March. The longest data set, for the Mississippi River at Vicksburg, demonstrates a similar direct relationship with the NAO Index from 1826 to 1969. After 1969, however, the relationship between discharge and the NAO Index is insignificant even though the range of Index values overlap for the two intervals. A breakpoint and rise in discharge ca. 1970 is consistent with well-documented land cover and land use changes occurring then that resulted in reduced evapotranspiration as homogenous cropping systems were established, and a higher percent of precipitation was routed into groundwater and baseflow. The Bonnet Carré Spillway at New Orleans, LA, is being opened more frequently to reduce flood threats as the river's stage increasingly reaches the threshold for opening it. Significant water quality impairments in the coastal zone will appear or be sustained with these openings. These data may be useful for climate change assessments through modeling or synthetic assessments in combination with other data sets.

Introduction

Variations in river discharges reflect changing climates and land use to create flooding and drought potentials, sustain or alter channel morphologies and navigation possibilities, and influence many other dimensions of the physical world. The Mississippi River drains the

design, data collection and analysis, decision to publish, or preparation of the manuscript.

Competing interests: The authors have declared that no competing interests exist.

largest watershed in North America including 41% of the conterminous US land area and two Canadian provinces; it is third, sixth, and eighth of the largest rivers in the world in terms of length, sediment yield, and discharge, respectively [1–2]. Droughts and floods are focus issues for farming enterprises occupying two-thirds of the watershed that produce 33 and 34% of the world's commercial yields of corn and soybeans, respectively [3]. The river is a national trade route, waste depository, and source of cooling and drinking water that has had some water quality improvements in the last half century [4]. Knowing the variabilities in discharge, and perhaps predicting them, is important.

Some of the variabilities in discharge have been documented and are known to be related to continental scale weather patterns fluctuating over decades. For example, discharges increased in the Upper Mississippi River watershed at 57 stations from 1940 to 1999 [5, 6], in midwestern USA watersheds from 1935 to 2014 [7], from 1940 to 2003 in the entire Mississippi River watershed [8] and from 1900 to 2018 [9]. These increases were related, in part, to variations in the position and intensity of the North Atlantic jet stream represented by the North Atlantic Oscillation (NAO) Index which records surface westerlies from the north Atlantic across Europe [10, 11]. Decade-long variations in seasonal rainfall and heat variations over the North Atlantic are linked with variations in the NAO, particularly during winter [12]. A pregnant question in consideration of future climate conditions is whether or not future variations in river discharge and NAO will retain the proportionality of previous years. Modeling outputs demonstrate quite a bit of uncertainty about these climate: discharge relationships which will become important to resolve. For example, the most recent Intergovernmental Panel on Climate Change [13] Report predicts increases in the Mississippi River discharge under five warming scenarios, but the 95% confidence level is many times larger than the predicted changes and so it includes the possibility of lower discharges [Fig 8.26 in 13].

Increased discharges imply increased flood potentials and so flood predictions are a related concern. Alfieri et al. [14] used 7 models to estimate global flood risk under 1, 2.5 and 4˚C warming scenarios which were exceeded by 2021, 2036 and 2082, respectively. The Mississippi River watershed was included but they did not specify predictions for flooding therein. They concluded that "even under the most optimistic warming scenario of 1.5˚C, we estimate a more than doubling of global flood risk as compared to 1976–2005." Their results contrast with those of Huang et al. [15] who used data from 1980 to 2001 for the Upper Mississippi River at Alton, IL, in 4 models to compare flood indices under 1.5, 2.0 and 3.0˚C climate change scenarios. They predicted that future annual floods will be less frequent and that flooding then will occur earlier in the year. There are, therefore, diverse climate model outcomes–both higher and lower–of future discharges in the Mississippi River watershed and sub-watershed regions that are at least partly based on disparate discharge record lengths. This situation is not unreasonable given the nascent efforts, uneven quality and quantity of data sets used, and their temporal length. The accuracy of model outputs might be further improved by using longer discharge records and ones that were about sub-watershed regions.

Here I assemble and analyze a unique data set of the annual average, minimum and maximum discharge data for three locations on the mainstem of the Mississippi River (Clinton, IA (41˚50'40"N; 90˚11'19"W), St. Louis (38˚37'37"N; 90˚11'58"W), and Vicksburg, MS (32˚ 21'10"N; 90˚52'40"W)), one on the Missouri River (Herman, MO (38˚42'15N; 91˚25'15"W), and one on the Ohio River (Louisville, KY (38˚15'10"N; 85˚45'30"W). The longest dataset is from Vicksburg, MS, which dates from 1817 to 2020. The annual variations of all three measures are discussed within the context of the NAO, and the influence of land use and dams. An example of a consequence of increasing river discharges downstream near New Orleans, LA, is the potential for more frequent use of the flood-preventive Bonnet Carré and Morganza Spillways in the lower Mississippi River and is discussed.

Methods

Description of the watershed

The Mississippi River watershed has six subbasins (Fig 1) whose land surface area is dominated by the Missouri River watershed (42%), which compares to the almost equal percentages of the Ohio, Upper Mississippi, and Arkansas River watersheds (sum = 45%); the remaining 14% of the watershed is almost equally divided between the Red River and Lower Mississippi River watersheds. In contrast to the total land area, which is highest in the Missouri River watershed, the Ohio River watershed provides the highest water discharge and amounts to an average 38% of the total volume followed by 19%, 13%, 13%, 11%, and 11% for the Upper Mississippi, Missouri, Lower Mississippi, Red and the Arkansas rivers, respectively [16]. The river discharge measurements discussed below are from five locations shown in Fig 1: 1) Clinton, IA, on the Upper Mississippi River, 2) Herman, MO, on the Missouri River, 3) St. Louis, MO, downstream of where waters from the Upper Mississippi River and Missouri River converge, 4) Louisville, KY, on the Ohio River upstream of where the Tennessee River joins it, and 5) at Vicksburg, MS, downstream of where the Ohio, Missouri, and Upper Mississippi River merge. The river is constrained by flood protection levees along most of its length and populated by revetments and locks, especially in the northern portion.

Floods from the Mississippi River, which can be significant throughout the watershed, became a prominent focus after the disastrous 1927 flood in the Lower Mississippi River Valley [17]. There are now 6095 km of embankments and floodwalls, including 3566 km along the main channel [18] that were supplemented by reticulated concrete cement 'mattress' revetments to protect the levees, and three diversions of the Mississippi River downstream of Vicksburg, MS (Fig 2). The tripartite Old River Control Structure complex, which is 40 km north of St. Francisville, LA, was completed in 1964 and expanded in 1994. It diverts (up to its capacity) 30% of the Mississippi River westward where it joins with the more modest flows of the Red

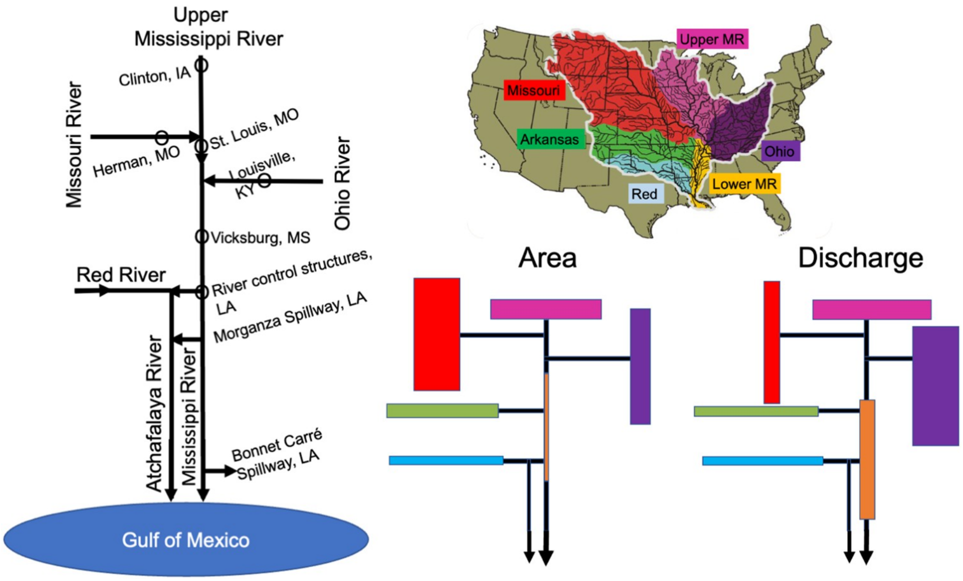

Fig 1. A schematic model of the Mississippi River watershed and major subbasins, comparative areas and discharge volumes and major water distributions with the five locations of the discharge measurements. The relative area and discharges are proportional amounts (expressed as a percent of the total) for 1973–1994. The baseline map of six subbasins is from the Mississippi Valley Division of the U.S. Army Corps of Engineers (mvd.usace.army.mil) at https://www.pinterest.com/pin/mississippi-valley-division-about-mississippi-river-commission-mrc--34.

https://doi.org/10.1371/journal.pone.0276513.g001

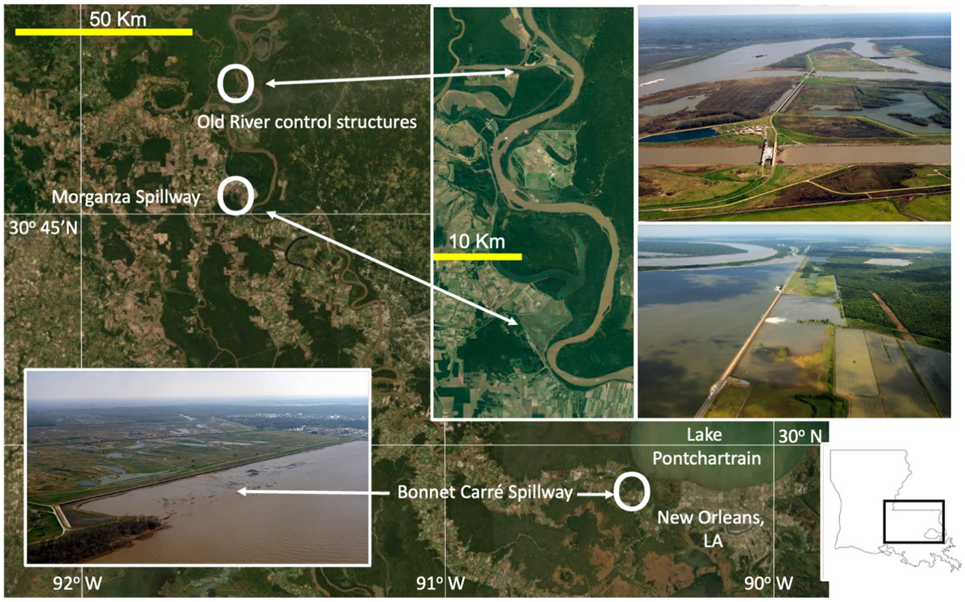

Fig 2. The three water control structures diverting water away from the main channel of the Mississippi River before it reaches New Orleans, LA. The most upstream is the Old River Control Structure that has three channels bringing river water westward where it joins the Red River to form the Atchafalaya River. The Morganza Spillway, located downstream, has been opened twice during extremely high flows. The Bonnet Carré Spillway brings water into Lake Pontchartrain located on the north shore of New Orleans, LA. The satellite imagery is from a one-meter resolution coastal Digital Orthophoto Quarter Quadrangle (DOQQ) imagery provided by the U.S. Geological Survey to the Atlas Depository (public access) at https://maps.ga.lsu.edu/doqq2008/. The spillway photographs are from the U. S. Army Corps of Engineers Digital Visual Library (public access) at http://images.usace.army.mil/.

https://doi.org/10.1371/journal.pone.0276513.g002

River which then becomes the Atchafalaya River that flows southward through the Atchafalaya Basin–an older main channel of the Mississippi River. The Morganza Spillway, completed in 1951, is a few kilometers farther south at Morganza, LA, and it was opened in 1973 and 2011 to reduce flood heights during exceptionally high river flows; it has a maximum discharge of 16,990 m^3 sec^{-1}. Another diversion on the upstream border of the New Orleans metropolitan area is through the Bonnet Carré Spillway; it brings Mississippi River water over the left descending bank and into Lake Pontchartrain on the northern shore of the City of New Orleans and has a maximum capacity of 7079 m^3 sec^{-1}. It was completed in 1931 and opened 16 times through 2021. The principal purpose of these diversions is to reduce flood inundations and to protect the City of New Orleans from catastrophic flood events. The Morganza Spillway is also intended to stabilize the main course of the Mississippi River so that it is not re-directed into the Atchafalaya River.

Bonnet Carré and Morganza Spillways

A perspective was developed of how increased discharges in the lower Mississippi River may affect the frequency of recent openings of the Bonnet Carré and Morganza Spillways. These three river diversions protect against a hypothetical maximum discharge event, the Project Design Flood, of 76,739 m^3 sec^{-1} at Vicksburg, MS, and 42,475 m^3 sec^{-1} at New Orleans, LA [19]. The Project Design Flood is a hypothetical "maximum probable" flood of the Mississippi River used by the United States Army Corps of Engineers to aid in the design and execution of flood protection in the Mississippi Valley [19]. The total maximum flow through the Old River Control Structure during this hypothetical maximum flow is 17,556 m^3 sec^{-1} which is 22% of

the Project Design Flood at Vicksburg, MS. The threshold for opening the Morganza Spillway is 42,000 m^3 sec^{-1} at the Mississippi River a few kilometers downstream of the Old River Control Structure but upstream of the Bonnet Carré Spillway; the predicted threshold for opening the Bonnet Carré at New Orleans is 35,396 m^3 sec^{-1}. If the discharge is anticipated to exceed the Bonnet Carré spillway maximum capacity (42,475 m^3 sec^{-1}), then the Morganza Spillway further upstream can divert an additional amount not to exceed 16,900 m^3 sec^{-1}. These thresholds are dependent on an average stage: discharge relationship among years that is not constant, and so it is the height of the water not the discharge that determines flood risk. The various discharge volume thresholds for diverting water are an approximation for actual conditions that are considerate of stage: discharge relationships and anticipated rainfall amounts. The 1973 flooding at St. Louis, MO, for example, was described as a 1-in-200 year flood event but was a 1-in-30 year discharge event because of channel confinement, riverbed erosion and sedimentation [20]. The opening of the Morganza Spillway in 1973 was done, in part, to relieve pressure on the Old River Control structures that had eroded at the spillway base during the same flood event. The effect of discharges by the projected increases in precipitation also are important in predicting future flows.

The 62-year maximum discharge of the Mississippi River from 1950 to 2021 was plotted with the thresholds along with a simple linear regression ± 99% confidence interval of the maximum annual discharge below the Old River Control structures. The intent was to visually compare fixed threshold amounts to previous and predicted future discharge volumes.

Discharge data

I used two sources of the daily river discharge data that have overlapping data records with annual data. The annual data at the five locations variously extend from as early as 1817 to 2021. The annual average, minimum, and maximum discharge from 1817 to 1953 on the Mississippi River main channel at Clinton, IA, St. Louis, MO, and Vicksburg, MS, the Missouri River at Herman, MO, and the Ohio River at Louisville, KY, were reported by the Mississippi River Commission [21]. These data were compared to the second dataset derived from the daily discharge records available at the United States Geological Survey (USGS) (this second set is available at the USGS website @ https://waterdata.usgs.gov for each state under the tabs for 'current conditions', then 'daily discharges', and then 'time series' tabs). All records are at least ninety-seven years in length. The ratio of the annual maximum/minimum and annual average/minimum in these daily data discharge measurements was calculated for each of the five locations.

I used Prism 9.0.0 software © 2020 (GraphPad Software, Inc., La Jolla, CA) for statistical analyses with significance at $p = 0.05$. The data were fit to a spline/Lowess analysis with 4 nodes and the inflection point identified where the discharge rates went from a declining to an increasing rate. A simple linear regression curve fit was run for the maximum discharge data at Vicksburg, MS, from 1970 to 2021 and for the years of overlapping annual discharges.

A three-year-running average for the annual average discharge at each of the five sites was constructed for 1970 to 2020. These averages were normalized to the mean discharge value for all years, so that the average = 1 for each of the five sites. Another dataset was constructed that included the normalized annual average and maximum discharge rates for 1940 to 2020, and the maximum: average discharge ratio; this ratio was intended to create an index for flooding each year in relationship to the average. Data for the annual minimum and maximum discharges at Vicksburg, MS, were separated by the month of occurrence and the frequency for each month was compared for the periods from 1817 through 1917, 1917 to 1969, and from 1970 to 2020.

North Atlantic Oscillation (NAO)

Discharges at the five stations were compared to two constructions of the North Atlantic Oscillation (NAO) Index downloaded from well-established climate data repositories. Both data sets have monthly data and one includes overlaps with all but the first seven years of the discharge data for Vicksburg, MS.

A monthly index for 1950 to 2021 is available from the National Oceanographic and Atmospheric Administration (NOAA) at https://www.cpc.ncep.noaa.gov/products/precip/CWlink/pna/nao.shtml that is the result of a Rotated Principle Component Analysis of the 500 mb atmospheric pressure between 20˚ and 90˚ N (https://www.cpc.ncep.noaa.gov/products/precip/CWlink/daily_ao_index/history/method.shtml) that was first described for the 700 mb pressure by Barnston and Livezey [22]. An exploratory analysis parsed the dataset used which was the average NAO Index for January, February and March of each year (NAO_{jfm}). These months were chosen because they precede or overlap months with the peak maximum river discharge for most years. A simple linear regression of a 3 year running average of the discharge at the five stations and the 3 month average of the NAO_{jfm} Index was halved into two 35 year intervals from 1950 to 1985, and from 1986 to 2020. I tested for differences in slopes between the two intervals using the Prism software.

A longer NAO_{jfm} Index dataset was for 1824 to 2020 (University of East Anglia Climate Research Unit; https://crudata.uea.ac.uk/cru/data/nao/nao.dat). This NAO Index is the difference between the normalized sea level pressure over Iceland, Gibraltar and the Azores [23]. I generated a simple linear regression equation of the three-year running average of the discharge at Vicksburg, MS, versus the three-year running average of the NAO_{jfm} Index for 1824 to 1969 and for 1970 to 2020. The beginning year 1970 was chosen for its 50 year length and because that is when indices of water quality such as alkalinity and nutrient concentrations began to increase sharply [9, 24].

Dams

Data from the 2018 National Inventory of Dams (https://nid.usace.army.mil/ords/f?p=105:1::::::) were assembled for 12 states with a portion of their territory in the Missouri River basin (Colorado, Illinois, Iowa, Kansas, Minnesota, Missouri, Montana, Nebraska, North Dakota, South Dakota, Wisconsin, and Wyoming) and 5 states in the Ohio River basin (Kentucky, Indiana, Ohio, Tennessee and West Virginia). These represent 31,214 dams in the Missouri River+Upper Mississippi River watersheds and 4,224 dams in the Ohio River watershed. There were an additional 1,910 dams in the Midwest region and 427 in the Ohio River region that had no construction date. The area included in these states is larger than the sub-basin area which will result in an overestimate of dams, but many dams are unreported because of their size, location and age. Thousands of mill dams, for example, are missing from the inventory of 90,000 dams throughout the US [25, 26]. Estimates of the 'normal storage' of dams began in the early 1800s and the cumulative volume was summed by year completed.

Results

Discharges

The overlap of the Mississippi River Commission and USGS data records ranged from 20 to 90 years; the Coefficient of Determination (R^2) = 0.99 and there was a slope of 0.99 for each of the five sites (S1 Fig). Those well-defined relationships give credibility to the robustly consistent efforts to estimate discharge.

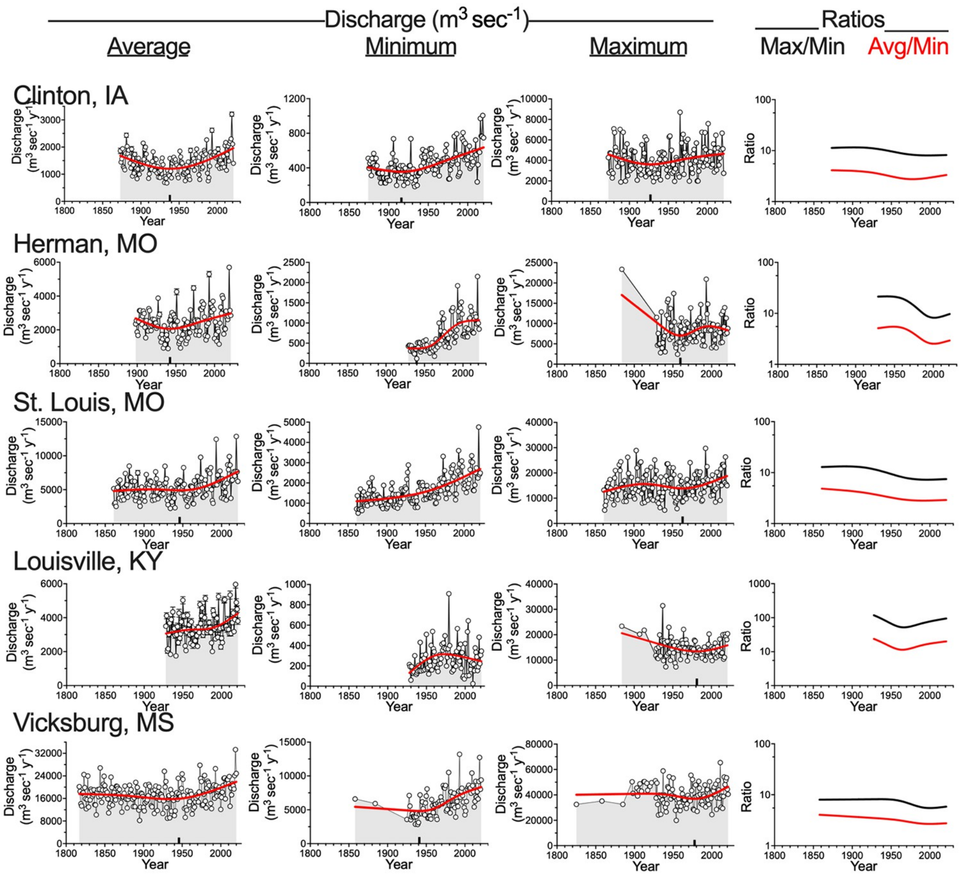

Fig 3. The annual average, minimum, and maximum discharge (m^3 sec^{-1}) and the ratios of the maximum/minimum (red line) and average/minimum (black line) at the five stations in the Mississippi River watershed. Clinton, IA, St. Louis, MO, and Vicksburg, MS, are on the main stem of the Mississippi River; Herman, MO, is on the Missouri River, and Louisville, KY, is on the Ohio River. The inflection point where a decrease becomes an increase is indicated by the small black bar on the horizontal axis.

https://doi.org/10.1371/journal.pone.0276513.g003

The annual average discharge at all five sites has been increasing in the last few decades with the peak in 2019 for all sites, but with unequal proportionalities between sites of the size of the 2019 peak relative to the average flow for that year (Fig 3). The inflection in the curve fit for four sites was in the year 1943.2 ± 2.2 (μ ± 1 SEM; n = 4) and there was no inflection point in the briefer data record at Louisville, KY. There were five peaks at Clinton, IA, and Herman, MO (1927, 1951, 1973 1993 and 2019) that are found downstream at St. Louis, MO, but not as conspicuously so at Vicksburg, MS. The peaks at Louisville, KY, were asynchronous with those at the other stations, except for in 2019. The annual average discharge at Vicksburg, MS, was 15% higher after 1970 than before 1970 ($p < 0.001$), but when compared between the same time intervals, the annual minimum and maximum discharges were not significantly different from each other (S2 Fig). The normalized change in the average discharge at Vicksburg, MS, from 1970 to 2020 was 4.48% decade^{-1}.

The minimum daily discharge in any year at all stations also increased with the exception of at Louisville, KY, and there was a peak minimum discharge in 2019 at all stations. There was no synchronicity of the minimum peak discharge at Louisville, KY, compared to at any of the other sites with the exception that the highest minimal discharge there was in 1979, which was

the time of the third highest peak at Vicksburg, MS. The annual minimum discharge at Vicksburg, MS, was 32% higher after 1970 than before 1970 ($p < 0.01$; S2 Fig).

The maximum daily discharge in any year increased at all stations for the last four decades with the inflection point occurring earliest at Clinton, IA (1927), the latest four decades ago at Louisville, KY (1981), and between 1960 and 1980 at the other three stations. The average inflection point for all five stations was 1956 ± 15 y (μ ± 1 SEM). The years of peaks are not always the same for the maximum annual flows as for the average annual flows. For example, the highest peak in the annual maximum discharge at Vicksburg, MS, was in 2011, but the highest average flow was in 2019; the highest maximum flow at Clinton, IA, was in 1965, but the highest average flow was in 2019. There was a high maximum peak in 1993 at Herman, MO, but no outstandingly high peak in 2019, whereas there was a 1993 peak in the average flow at Herman, MO, but an even higher one in 2019. The annual maximum discharge at Vicksburg, MS, was not significantly higher after 1970 than before 1970 ($p = 0.82$; S2 Fig). The normalized change in the maximum discharge at Vicksburg, MS, from 1970 to 2020 was 2.31% decade^{-1} (S3 Fig).

The ratio of the annual maximum: annual minimum and the annual average: annual minimum discharges declined at all sites except for at Louisville, KY (Fig 3). The declines illustrate how the annual minimum discharge is rising disproportionately faster than the annual average or annual maximum discharge. The normalized change in the annual maximum: annual average discharge at Vicksburg, MS, from 1970 to 2020 was -2.30% decade^{-1}.

Monthly peak frequency

There was no apparent difference for when either the minimum or maximum annual discharge occurred by month at Vicksburg, MS, for the first 100 years (1817–1916). But the monthly minimum for the last 50 years (1970–2019) came earlier in the fall compared to in 1817–1916 (Fig 4). There were two peaks of maximum flood height in the spring as a result of the lower frequency of maximum flood occurrences in April. In other words, after 1970, the previously consistent annual spring peak in discharge was split into two peaks, with a slightly earlier chance (< 10%) of the maximum flow occurring one month earlier from 1970 to 2020.

Discharge synchronicities

The three-year averaged annual discharge at the five locations showed similar rises and falls (Fig 5). The R^2 for a simple linear regression of the annual average discharge at Vicksburg, MS, versus at the other four locations ranged from 0.27 to 0.47 and $p < 0.0001$ for all four regressions (not shown).

NAO_{jfm} Index

The discharges at all sites were directly related to the NOAA derived NAO_{jfm} Index for 1950 to 2020, and there were no differences in slope between the two thirty-five year intervals from 1950 to 1985 and from 1986 to 2020 at Clinton, IA, Louisville, KY, and Herman, MO (Fig 6). The discharge at St. Louis, MO, and Vicksburg, MS, were directly related to the NAO_{jfm} Index for the interval from 1950 to 1985, but not from 1986 to 2020. The distribution of discharge points for the two intervals overlapped about 50% of the range along the x-axis, illustrating the higher NAO_{jfm} Index in the last 35 years compared to the first 35 years. The R^2 ranged from 0.17 to 0.32.

The discharge at Vicksburg, MS, was directly related to the longer University of East Anglia derived NAO_{jfm} Index that included 1824 to 1969, but not to the NOAA-derived NAO_{jfm} Index for 1970 to 2020 (Fig 7). The slopes of the two equations were different ($F = 7.692$, $DFd = 190$; $p = 0.0061$) and the distribution of yearly discharge points along the x-axis for the two time periods overlapped for 190 of 194 years.

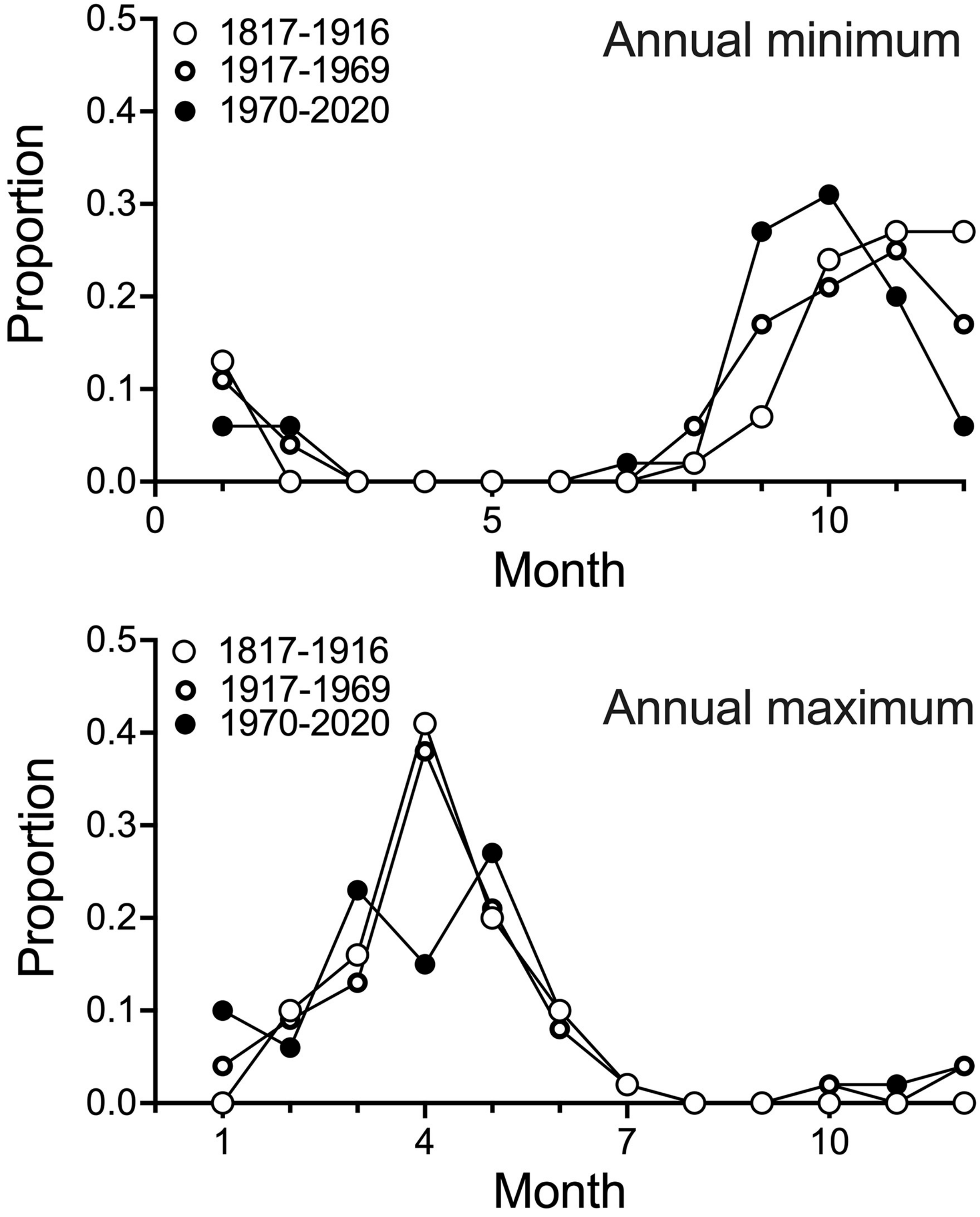

Fig 4. The monthly proportion during the year for the annual minimum (upper panel) and maximum (lower panel) discharge (m^3 sec^{-1}) at Vicksburg, MS, from 1817 to 1916, from 1917 to 1969, and from 1970 to 2020.

https://doi.org/10.1371/journal.pone.0276513.g004

Dam volumes

The storage capacity of dams in the Missouri and Upper Mississippi River watersheds climbed rapidly after the 1930s and reached 95% of their 2018 capacity by 1975 (Fig 8). The dam data with missing years had a cumulative storage volume of < 0.5% of the total volume in 2018.

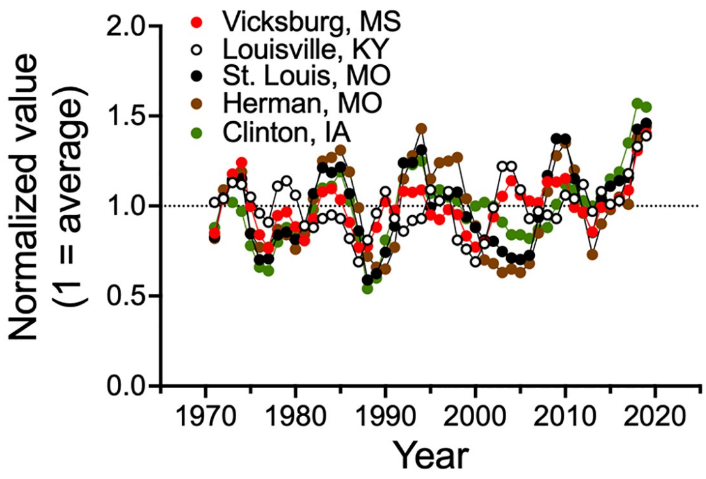

Fig 5. The three-year average discharge of the Mississippi River at the five stations, normalized to the average discharge at each for 1970 to 2021.

https://doi.org/10.1371/journal.pone.0276513.g005

The volume difference between the 'normal capacity' and the 'maximum capacity' (32%) is large enough to hold more than a month's average discharge of the Mississippi River at Vicksburg, MS.

Bonnet Carré Spillway

A simple linear regression of the maximum discharge at New Orleans and year for from 1940 to 2020 was Y = 68.67*X– 105,536 (F = 5.4, Dfd = 80, and $p = 0.06$; Fig 9). This is equivalent to an average 2.31% decade^{-1} increase in maximum discharge. If the current rates of increase continue, and assuming no change in the stage: discharge relationship, then within 20 years the irregular openings of the Bonnet Carré and Morganza Spillway will occur once every other year, on average. Similarly, the predicted discharge of the Project Design Flood will be within the 99% confidence limit of the maximum Project Flood design within six to seven decades.

Discussion

This is a coarse review of changing discharges throughout the world's third largest river watershed. The discharges at the five stations that were investigated, particularly at Vicksburg, MS, integrate many aspects of the heterogeneous climatic and hydrologic parameters occurring at the regional and local scale. A more general use of these long-term data records may facilitate stronger model developments. There are four consistencies in the aggregate that are discussed below, along with a brief description of consequences for flooding at New Orleans and for coastal ecosystems.

Increase in Mississippi River discharge

The data on the average annual discharge at Vicksburg, MS starts in 1817, and the discharge increased 4.48% decade^{-1} from 1940 to 2020. The average annual discharge was increasing at the other four sites as well, although the data records of St. Louis, MO, Clinton, IA, Herman, MO, and Louisville, KY, are shorter, starting in 1861, 1873, 1898 and 1932, respectively. The shortest record is for Louisville, KY, and the discharge there also increased over the total data record. The initial rise in the average discharge at Vicksburg, MS, began in 1943, and could have been related to many factors, but its continuation through to 2020 was not due to changes

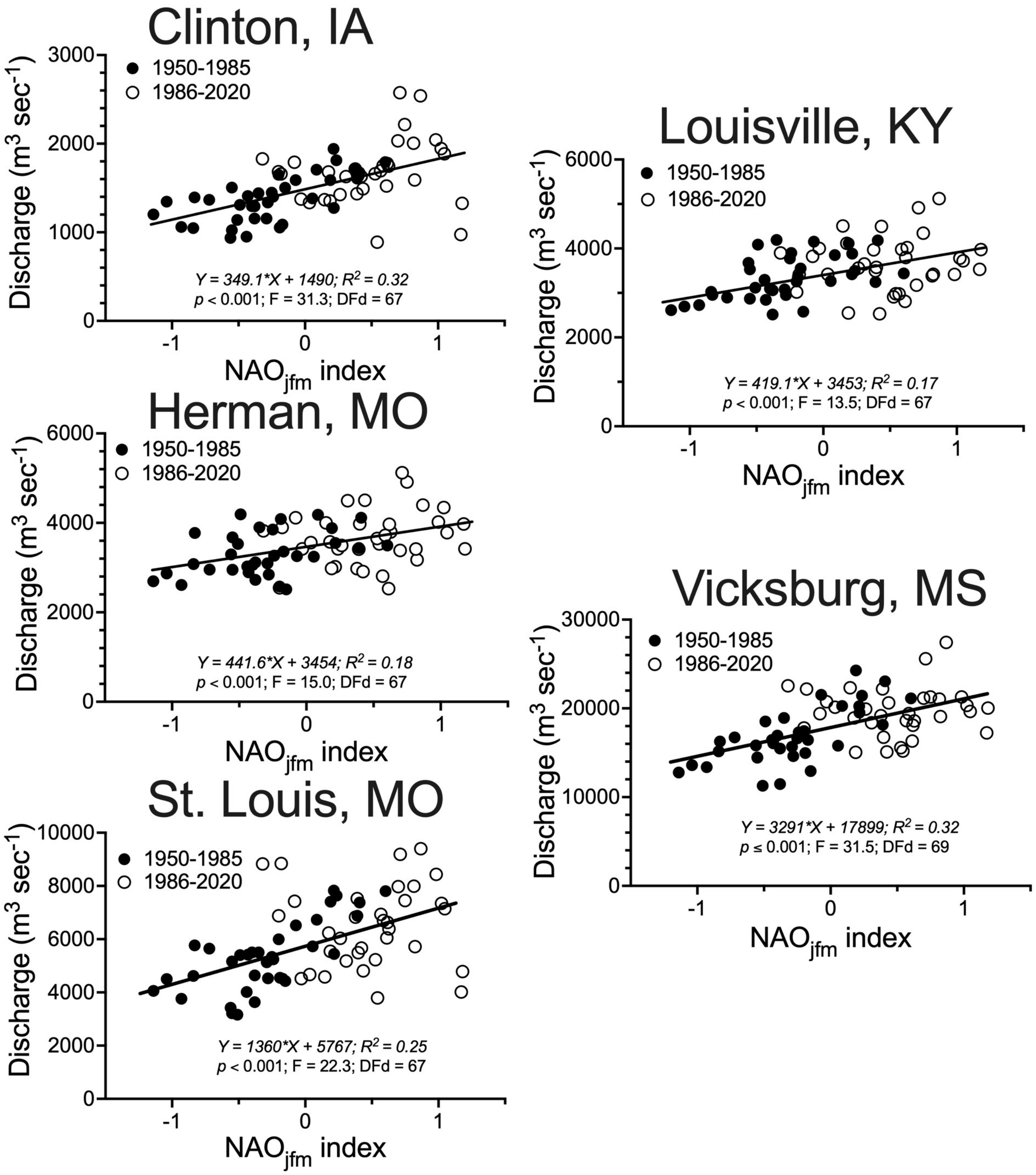

Fig 6. The relationship between the three year running averages for the discharge at the five stations versus the North Atlantic Oscillation Index for January, February and March (NAO_{jfm}), separately analyzed for 1950 to 1985 (filled circles) and 1986 to 2021 (open circles).

https://doi.org/10.1371/journal.pone.0276513.g006

co-related to the NAO_{jfm} Index. In fact, the relationship between the average annual discharge and the NAO_{jfm} seems to have broken down in the early 1970s. Despite incomplete synchronicity, the ups and downs of discharge at the five stations move together and within relatively similar ranges relative to their average discharge rates.

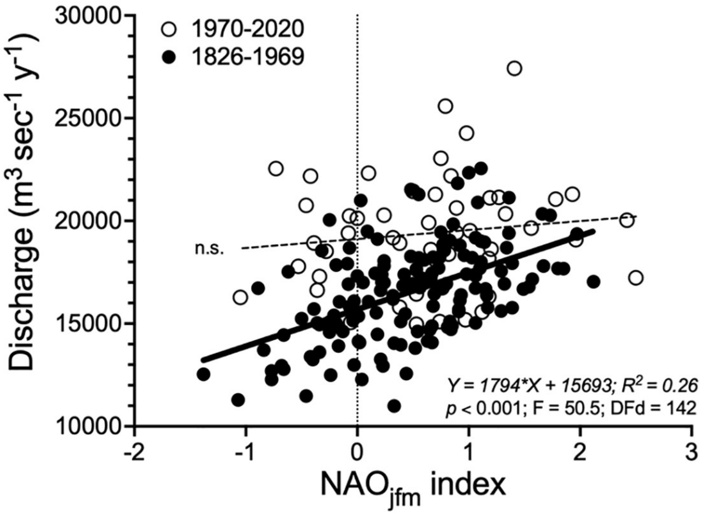

Fig 7. The simple linear regression relationship between the three year running averages for the annual discharge of the Mississippi River at Vicksburg, MS, versus the North Atlantic Oscillation (NAO) Index for January, February and March. The data are separated for between 1826 to 1969 (filled circles) and 1970 to 2020 (open circles). The dark broad line is significant; the dotted line is not significant (n.s.).

https://doi.org/10.1371/journal.pone.0276513.g007

These results complement Shilling and Libra's [27] incipient analyses of the temporal changes in discharge in 13 Iowa watersheds that had increases in the annual discharge, annual minimum, and the percentage of the annual baseflow in the total discharge from 1940 to 2003. Zhang and Schilling [8], Schilling et al. [28], and others showed how the shift from perennial plants to annuals, principally to soybean and corn, resulted in reduced evapotranspiration rates which led to more groundwater storage and runoff in the baseflow that increased discharge rates. Row crops, in general, do not retain water as well as deep-rooted perennials.

It should be recognized that sub-sections of the watershed are predicted to have sustained droughts and higher temperatures this century. A notable one is in the Southwest Missouri River subbasin that the Herman, MO, drainage station includes [29, 30]. Cook et al. [29]

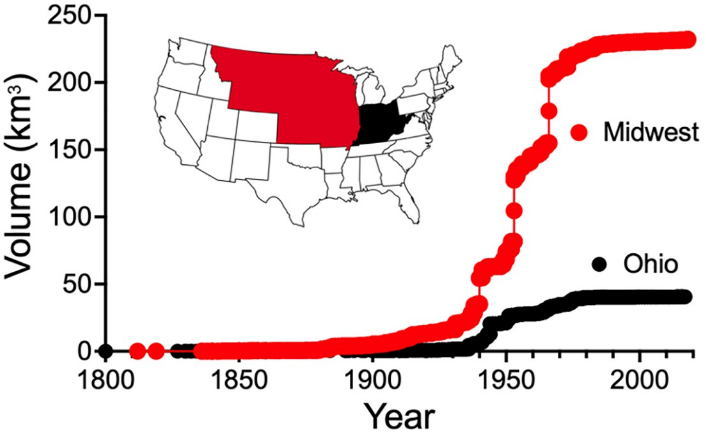

Fig 8. The volume of water stored behind dams and reservoirs in twelve states in the Midwest (Missouri River and Upper Mississippi River watersheds in red) and five states including the Ohio River watershed (in black).

https://doi.org/10.1371/journal.pone.0276513.g008

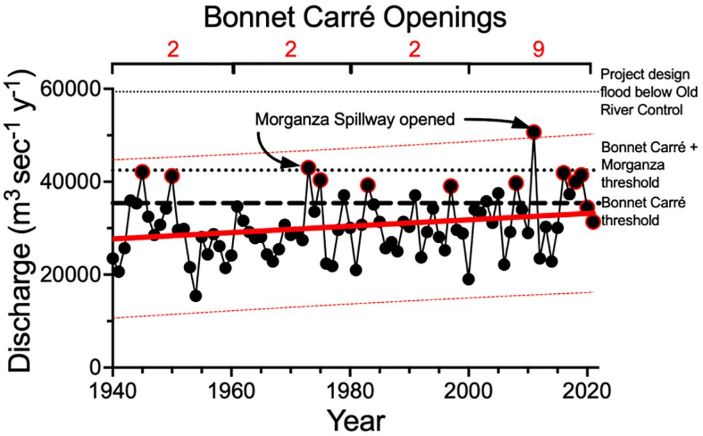

Fig 9. The maximum annual Mississippi River discharge at Vicksburg, MS, within the context of a linear regression of the discharge trend from 1940 to 2021. The thick red line is the predicted discharge, and the dashed red lines are the ± 99% confidence limits; the capacity of the US Corps of Engineers 'Project Design Flood' below the Old River Control Structure is the fine dotted line at the top of the figure. The openings of the Bonnet Carré and Morganza spillways upstream of New Orleans, LA, (black circles with red borders) are indicated with the thresholds for opening each. The Bonnet Carré Spillway was opened twice in both 2019 and 2021.

https://doi.org/10.1371/journal.pone.0276513.g009

modeled future emissions scenarios of the consequences of moderate and high Representative Concentration Pathways for a radiative forcing of 4.5 and 8.5 Watts m^2; they predicted that soil moisture would decline sharply around 2020 and that the area will be the driest in the past 1100 years by the end of this century.

Precipitation is not proportional to discharge

Precipitation has been increasing in the last 100 years in the Mississippi River watershed [31], but there is not a 1: 1 correspondence between precipitation and discharge; heterogeneity within watersheds will yield different outcomes than for the whole watershed. The percentage of precipitation that becomes discharge is modified by evapotranspiration, radiation, cloud cover, and both soil and air temperature. Evaporation from reservoirs is a small amount and does not contribute to the water budget changes. Milly and Dunne [32] estimated that evapotranspiration in the Mississippi River watershed from 1949–1997 was 649 mm compared to 835 mm of precipitation (78%) and that only 3 mm were lost to groundwater and reservoirs leaving 187 mm for river discharge. Minor changes in these other factors could, therefore, have a significant effect on discharge rates. Evaporation increases with higher temperature and so runoff is reduced if there are no changes in precipitation. Evaporation is dependent on many more factors than precipitation, including wind, humidity, and radiation. Roderick et al. [33], for example, demonstrated that evapotranspiration over land does not increase as fast as precipitation in their global model that did not assume changes in land cover. Xu et al. [34] found that 55 streams in the Midwest that were without dams and reservoirs had precipitation increases of about 8 percent, but that potential evapotranspiration decreased by 2%. Yet the conversion of forested land to croplands results in less evapotranspiration because of the reduced vegetative cover, which raises runoff that artificial drainage increases further [8, 11, 35, 36]. Thus, precipitation was a stronger predictor of streamflow in forested watershed than in intensely farmed watersheds of the Upper Mississippi River watershed [37] which is consistent with Xu et al.'s [34] conclusion that land use change contributed twice as much as climate change to stream discharge increases in the Midwest.

Dams

Dam capacity increased rapidly from the 1940s to the 1970s, and reduced surges in upstream discharges which may partly explain why the annual maximum discharge: annual average discharge ratio has been decreasing–the maximum discharge upstream becomes spread out over a longer time period downstream. Graf [38] found that compared to upstream of dams, that the regulated reaches of 36 rivers had peak discharges that were lower by an average 67%, the annual maximum: mean flows were reduced by 60%, and the timing of minimum and maximum flows advanced by up to 6 months. Preparations for anticipated flooding may have stimulated management in the form of preemptive drawdowns. But dams will eventually fill in with sediments and some are being removed [39]. A small amount of evapotranspiration from dams occurs that Milly and Dunn [32] estimated to be 1 mm of the 835 mm of precipitation into the Mississippi River water.

NAO_{jfm}

The relationship between the annual river discharge over the last 50 years and the 194 year long NAO_{jfm} Index record from the University of East Anglia is not robust—the cloud of points for discharge from 1970 to 2020 are not aligned with the regression line for the previous 144 years. This difference is consistent with either a changing NAO_{jfm} Index or the relationship of discharge to it. But the range of the NAO_{jfm} Index has not changed over the last two centuries, and so the role of changing temperature, cloud cover, net radiation, evapotranspiration and land use appears to have a more prominent role influencing the higher discharge rates.

Bonnet Carré Spillway openings

The Bonnet Carré opened 16 times since 1931, and 4 times from 2019 to 2021. Its more frequent use is occurring as the statistically-defined maximum river discharge rises and approaches the threshold discharge rates for its use every other year (on average), which will occur within two decades at the present rate of increase. The maximum annual discharge exceeded the 99% Confidence Interval (CI) in 2011, and the 99% CI will overlap the discharge threshold for using the Morganza Spillway before the end of the century if the rate of increase since 1950 continues. That means that a flood then could exceed the Project Flood capacity. Expanding diversion and spillway capacities could delay the inevitable if current rises in the discharge continue, and the expansion costs would be non-trivial. To assume that there will be a continued increase in the discharge rate is problematic in the sense that the past is not the future; but it is certain that change is inevitable—the discharge rate may decrease but it could also continue to increase at its present rate or even accelerate. For the last 70 years the total annual discharge rates have been increasing about twice as fast as the maximum discharge rates because the amount of water in the maximum relative to the total discharge is declining at about half the rates of discharge increases. What this implies is that flood reduction efforts may be having an effect but are reducing only half of the rise in the maximum flood discharges. Ignoring the present increase in the discharge rates of the Mississippi River has consequences for the City of New Orleans and Morgan City at the end of the Atchafalaya Basin.

Coastal consequences

A more frequent opening of the Bonnet Carré will fill Lake Pontchartrain more often and more fully and the lake will then eutrophy because of the river's relatively high nitrate and phosphate concentration and the lake's slow turnover time (1.78% day^{-1}). The 1997 opening of

the Bonnet Carré Spillway, for example, resulted in the formation of an algal bloom with a maximum 855 μg Chl *a* L^{-1} in the middle of the lake [40]. Elevated levels of hepatotoxins were measured during the peak of the bloom that followed the 1997 opening [41]. Oyster harvests decline after salinities are lowered throughout the Gulf of Mexico estuaries [42], and the 2019 opening was followed by increased mortalities of the bottlenose dolphins (*Tursiops truncatus*) (https://www.fisheries.noaa.gov/national/marine-life-distress/2019-bottlenose-dolphin-unusual-mortality-event-along-northern-gulf)—a marine protected animal (Marine Mammal Protection Act and Endangered Species Act). Furthermore, the largest hypoxic area in the western Atlantic forms where the Mississippi River debouches into the Gulf of Mexico [43]. The main factor determining its size in summer is the river's nitrogen load in May [44], which is a combination of the nitrogen concentration in the river and its discharge. The nitrate concentration in tiles is higher than in water draining from fields without tiles [45] and model simulations indicate that nitrogen losses increase at a disproportionately faster rate than corn production as fertilization rates increase within the now commonly applied rates in tiled farm fields [46]. The implication is that higher precipitation in the future will sustain, if not enhance, the current nitrate loading rates under current tiling practices, not reduce them.

Conclusions

A two-century long dataset was compiled for the annual average, minimum, and maximum discharges at five stations in the Mississippi River watershed. These data may be useful for climate change assessments through modeling or synthetic assessments with other data sets. These three discharge metrics are increasing throughout the watershed in the last few decades, and the minimum annual discharge is increasing faster than either the maximum or average discharge. Whether or not those increases continue is important to monitor and interpret for a variety of reasons. Variations in discharge at Vicksburg, MS, before the 1970s are related to the air pressure differentials representing atmospheric forcing factors, but that was not the case after the 1970s; this result is consistent with the increasing influences from human perturbations affecting the percentage of precipitation that becomes discharge, its routing into baseflow, and seasonal distributions. The Bonnet Carré Spillway at New Orleans, LA, is being opened more frequently as the rise in the river's discharge reaches the threshold for opening it. Significant water quality impairments in the coastal zone will be hastened as a result if increases continue.

Belt [20] opened his outstanding analysis of the 1973 flood at St. Louis, MO, with a quote from Horace: "Naturam expelles furca, tamen usque recurret" ("Drive nature off with a pitchfork, nevertheless she will rush back."—Epistulae 1, 10, 24). The same conclusion could be considered in light of the human-caused changes illustrated using these river discharge metrics.

Supporting information

S1 Fig. Comparison of annual discharges of the Mississippi River at Vicksburg, MS, reported by the Mississippi River Commission (2007) and the USGS (https://waterdata.usgs.gov). The number of overlapping years ranges from 20 to 90 years and the Coefficient of Determination (R^2) = 0.99 and have a slope of 0.99 in all data sets.
(TIFF)

S2 Fig. Violin plots of discharges before 1970 and after 1969 at Vicksburg for the annual averages, minimums, and maximum. The mean and upper and lower quartiles are shown for each pair of sets. The probability of differences is shown for each of the three pairs. The t-test was for an unpaired parametric test with Welch's correction: Average: F = 4.165, Dfd = 152;

Minimum: F = 2.175, Dfd = 44; Maximum: F = 1.065, Dfd = 58.
(TIFF)

S3 Fig. The normalized changes in the annual average and maximum discharge at Vicksburg, LA, and the ratio of the Maximum:Average, where 1 = the average for 1940 to 2020. The equations are: 1) Average discharge: Y = 0.004483*X—7.876; F = 18.2, R^2 = 0.19, $p < 0.001$; 2) Maximum discharge: Y = 0.002309*X—3.572; F = 5.47, R^2 = 0.06, $p = 0.02$; 3) Maximum:Average: Y = -0.002297*X + 5.548; F = 9.72, R^2 = 0.11, $p < 0.01$. The slopes are different from each other. The average discharge is rising at 4.48% decade^{-1}, but the maximum discharge is rising 2.31% decade^{-1}. The difference can be attributed to the lower amount of the average discharge that becomes a maximum discharge, probably because dams and reservoirs reduce the height of peak discharges.
(TIFF)

Acknowledgments

The data collection was done by thousands of public servants over two centuries in the dim light of faint praise; they deserve our gratitude. This data harvesting and analysis was supported by the NOAA Coastal Ocean Program MULTISTRESS Award No. NA16OP2670 to Louisiana State University and NSF Award Number (FAIN) 2103843: Large-scale CoPe Hub: Rising Voices, Changing Coasts: The National Indigenous and Earth Sciences Convergence Hub. Lauris Hollis, Julie Torres and Chris Swarzenski are thanked for reviewing a draft manuscript. The river discharge data assembled are available at the Dryad repository at https://doi.org/10.5061/dryad.1jwstqjzb.

Author Contributions

Conceptualization: R. Eugene Turner.

Data curation: R. Eugene Turner.

Formal analysis: R. Eugene Turner.

Funding acquisition: R. Eugene Turner.

Investigation: R. Eugene Turner.

Methodology: R. Eugene Turner.

Project administration: R. Eugene Turner.

Resources: R. Eugene Turner.

Visualization: R. Eugene Turner.

Writing – original draft: R. Eugene Turner.

Writing – review & editing: R. Eugene Turner.

References

1. Milliman JD, Meade R. World-wide delivery of river sediment to the ocean. J Geol. 1983; 91: 1–21. https://doi.org/10.1086/628741
2. Best J. Anthropogenic stressors on the world's big rivers. Nat Geosci. 2019; 12: 7–21. https://doi.org/10.1038/s41561-018-0262-x
3. FAO (Food and Agriculture Organization of the United Nations). FAO Statistical Databases [accessed 2021 November 26]. http://faostat.fao.org. 2021.

4. Turner RE. Declining bacteria, lead, and sulphate, and rising pH and oxygen in the lower Mississippi River. Ambio. 2021; 50: 1731–1738. https://doi.org/10.1007/s13280-020-01499-2 PMID: 33550571

5. Lins HF, Slack JR. Streamflow trends in the United States. Geophys Res Lett. 1999; 26: 227–230. https://doi.org/10.1029/1998GL900291

6. Lins HF, Slack JR. Seasonal and regional characteristics of US streamflow trends in the United States from 1940 to 1999. Phys Geogr. 2005; 26: 489–501. https://doi.org/10.2747/0272-3646.26.6.489

7. Kelly SA, Takbiri Z, Belmont P, Foufoula-Georgiou E. Human amplified changes in precipitation-runoff patterns in large river basins of the Midwestern United States. Hydrol Earth Syst Sci. 2017; 21: 5065–5088. https://doi.org/10.5194/hess-21-5065-2017

8. Zhang YK, Schilling KE. Increasing streamflow and baseflow in Mississippi River since the 1940s: Effect of land use change. J Hydrol. 2006; 324: 412–422. https://doi.org/10.1016/j.jhydrol.2005.09.033

9. Raymond PA, Hamilton SK. Anthropogenic influences on riverine fluxes of dissolved inorganic carbon to the ocean. Limnol Oceanogr Lett. 2018; 3: 143–155, https://doi.org/10.1002/lol2.10069

10. Hurrell JW. Decadal trends in the North Atlantic Oscillation: regional temperatures and precipitation. Science. 1995; 269: 676–679. https://doi.org/10.1126/science.269.5224.676 PMID: 17758812

11. Frans C, Estanbulluoglu E, Mishra V, Munoz-Arrioloa R, Lettensaier DP. Are climatic or land cover changes the dominant cause of runoff trends in the Upper Mississippi River Basin? Geophys Res Lett. 2013; 40: 1104–1110. https://doi.org/10.1002/grl.50262

12. Chelliah M, Bell GD. Tropical multidecadal and interannual climate variability in the NCEP–NCAR reanalysis. J Climate. 2004; 17: 1777–1803.

13. IPCC 2021. Climate Change, 2021: The Physical Science Basis. Contribution of Working Group I to the Sixth Assessment Report of the Intergovernmental Panel on Climate Change. Cambridge: Cambridge University Press: 2021.

14. Alfieri L, Bisselink B, Dottori F, Naumann G, de Roo A, Salamon P, Wyser K, Feyen L. Global projections of river flood risk in a warmer world. Earth's Future. 2017; 5: 171–182. https://doi.org/10.1002/2016ef000485

15. Huang S, Kumar R., Rakovec O., Aich V., Wang X., Samaniego L., Liersch S., and Krysanova V. Multi-model assessment of flood characteristics in four large river basins at global warming of 1.4, 2 and 3.0 K above the pre-industrial level. Environ Res Lett. 2018; 13: 124005. https://doi.org/10.1088/1748-9326/aae94b

16. Turner RE, Rabalais NN. Suspended sediment, C, N, P, and Si yields from the Mississippi River Basin. Hydrobiologia. 2004; 511, 79–89. https://doi.org/10.1023/b:hydr.0000014031.12067.1a

17. Barry JM. Rising Tides: The Great Mississippi Flood of 1927 and How it Changed America. New York: Simon and Schuster; 1998.

18. United States Army Corps of Engineers. Mississippi River and Tributaries Project Levee System evaluation report for the National Flood Insurance Program. Mississippi Valley Division, U.S. Corps of Engineers [accessed 2021 November 27]. https://www.mvd.usace.army.mil/Portals/52/docs/MRC/LeveeSystem/MRT_levee_system_eval_report_for_NFIP.pdf. 2014.

19. Mississippi River Commission. The Mississippi River and Tributaries Project: Controlling the Project Flood 2007. Information Paper [accessed 2021 February 03]. 2007. https://www.mvd.usace.army.mil/Portals/52/docs/Controlling%20the%20Project%20Flood%20info%20paper.pdf.

20. Belt CM. The 1973 flood and man's constriction of the Mississippi River. Science. 1975; 189: 681–684. https://doi.org/10.1126/science.189.4204.681 PMID: 17792526

21. Mississippi River Commission. 1955. Annual maximum minimum and mean discharges of the Mississippi River to 1953. Vicksburg, MS. U.S. Army Corps of Engineers.

22. Barnston AG, Livezey RE. Classification, seasonality and persistence of low-frequency atmospheric circulation patterns. Mon Weather Rev. 1987; 115: 1083–1126. https://doi.org/10.1175/1520-0493(1987)115<1083:csapol>2.0.co;2

23. Jones PD, Jónsson T, Wheeler D. Extension to the North Atlantic Oscillation using early instrumental pressure observations from Gibraltar and South-West Iceland. Int J Climatol. 1997; 17: 1433–1450. https://doi.org/10.1002/(sici)1097-0088(19971115)17:13<1433::aid-joc203>3.0.co;2-p

24. Turner RE, Rabalais NN. Linking landscape and water quality in the Mississippi River Basin for 200 years. BioScience. 2003; 53: 563–572. https://doi.org/10.1641/0006-3568(2003)053[0563:llawqi]2.0.co;2

25. Smith SV, Renwick WH, Bartley JD, Buddemeier RW. 2002. Distribution and significance of small, artificial water bodies across the United States landscape. Sci Total Environ. 2002; 299: 21–36. https://doi.org/10.1016/s0048-9697(02)00222-x

26. Walter RC, Merritts DJ. Natural streams and the legacy of water-powered mills. Science. 2008; 319: 299–304. https://doi.org/10.1126/science.1151716 PMID: 18202284

27. Schilling KE, Libra RD. Increased baseflow in Iowa over the second half of the 20th Century. J Am Wat Resour Assoc. 2003; 39: 851–860.

28. Schilling KE, Jha MK, Zhang Y-K, Gassman PW, Wolter CF. Impact of land use and land cover change on the water balance of a large agricultural watershed: Historical effects and future directions. J Water Resour Res. 2008; 44: W00A09. https://doi.org/10.1029/2007wr006644

29. Cook BI, Ault RR, Smerdon JE. Unprecedented 21st century drought risk in the American Southwest and Central Plains. 2015; Sci Adv. 1: e1400082. https://doi.org/10.1126/sciadv.1400082 PMID: 26601131

30. Martin JT, Pederson GT, Woodhouse CA, Cook ER, McCabe GJ, Anchukaitis KJ, Wise EK, Erger L, Dolan PJ, McGuire M, Gangopadhyay S, Chase KJ, Littell JS, Gray ST, George S, Friedman JM, Sauchyn DJ, St-Jacques J-M, King J. Increased drought severity tracks warming in the United States' largest river basin. Proc Natl Acad Sci USA. 2020; 117(21): 11328–11336. https://doi.org/10.1073/pnas.1916208117 PMID: 32393620

31. Peterson TC, Heim RR Jr, Hirsch R, Kaiser DP, Brooks H, Diffenbaugh NS, Dole RM, Giovannettone JP, Guirgui K, Karl TR, Katz RW, Kunkel K, Lettenmaier D, McCabe GJ, Paciorek CJ, Ryberg KR, Schubert S, Silva VBS, Stewart BC, Vecchia AV, Villarini G, Vose RS, Walsh J, Wehner M, Wolock D, Wolter K, Woodhouse CA, Wuebbles DA. Monitoring and understanding changes in heat waves, cold waves, floods, and droughts in the United States: state of knowledge. Bull Am Meterol Soc. 2013; 94: 821–834. https://doi.org/10.1175/BAMS-D-12-00066.1

32. Milly PCD, Dunne KA. Trends in evaporation and surface cooling in the Mississippi River basin. Geophys Res Lett. 2001; 28: 1219–1222. https://doi.org/10.1029/2000gl012321

33. Roderick ML, Sun F, Lim WH, Farquhar GD. A general framework for understanding the response of the water cycle to global warming over land and ocean. Hydrol Earth Syst Sci. 2014; 18: 1575–1589. https://doi.org/10.5194/hess-18-1575-2014

34. Xu X, Scanlon BR, Schilling KE, Sun A. Relative importance of climate and land surface changes on hydrologic changes in the US Midwest since the 1930s: Implications for biofuel production. J Hydrol. 2013; 497: 110–120. https://doi.org/10.1016/j.jhydrol.2013.05.041

35. Blann KL, Anderson JL, Sands GR, Vondracek B. Effects of agricultural drainage on aquatic ecosystems: a review. Critical Rev Envir Sci Tech. 2019; 39: 11: 909–1001. https://doi.org/10.1080/10643380801977966

36. Schilling KE, Chan KS, Liu H, Zhang YK. Quantifying the effect of land use land cover change on increasing discharge in the Upper Mississippi River. J Hydrol. 2010; 387: 343–345. https://doi.org/10.1016/j.jhydrol.2010.04.019

37. Raymond PA, Oh N-H, Turner RE, Broussard W. Anthropogenic enhanced fluxes of water and carbon from the Mississippi River. Nature. 2008; 451: 449–452. https://doi.org/10.1038/nature06505 PMID: 18216851

38. Graf WL. Downstream hydrologic and geomorphic effects of large dams on American rivers. Geomorphology. 2006; 79: 336–360. https://doi.org/10.1016/j.geomorph.2006.06.022

39. Foley MM, Magilligan FJ, Torgersen CE, Major JJ, Anderson CW, Connolly PJ, Wieferich D, Shafroth PB, Evans JE, Infante D, Craig LS. Landscape context and the biophysical response of rivers to dam removal in the United States. PLos One. 2017; 12: 0180107. https://doi.org/10.1371/journal.pone.0180107 PMID: 28692693

40. Turner RE, Dortch Q, Rabalais NN. Inorganic nitrogen transformations at high loading rates in an oligohaline estuary. Biogeochemistry 2004; 68: 411–423. https://doi.org/10.1023/b:biog.0000031039.56794.29

41. Dortch Q, Peterson TD, Acheé S. The 1997 algal bloom in Lake Pontchartrain. In: *Effects of the 1997 Bonnet Carré Opening on Nutrients and Phytoplankton in Lake Pontchartrain*. Turner RE, Dortch Q., and Rabalais NN., Eds. Lake Pontchartrain Basin Foundation: Metairie, LA, USA, pp. 6.1–6.20. 1999.

42. Turner RE. Will lowering estuarine salinity increase Gulf of Mexico oyster landings? Estuar Coast. 2006; 29: 345–352. https://doi.org/10.1007/bf02784984

43. Rabalais NN, Turner RE. Gulf of Mexico hypoxia: past, present, and future. Limnol Oceanogr Bull. 2019; 28: 117–124. https://doi.org/10.1002/lob.10351

44. Turner RE, Rabalais NN, Justić D. Predicting summer hypoxia in the northern Gulf of Mexico, redux. Mar. Pollut. Bull. 2012; 64: 318–323. https://doi.org/10.1016/j.marpolbul.2011.11.008 PMID: 22153907

45. Hatfield JL, McMullen LD, Jones CS. Nitrate-nitrogen patterns in the Raccoon River Basin related to agricultural practices. J Soil Water Conserv. 2009; 64: 190–199.

46. Malone RW, Mab L, Heilman P, Karlen DL, Kanwar RS, Hatfield JL. Simulated N management effects on corn yield and tile-drainage nitrate loss. Geoderma 2007; 140: 272–283.
